# Microbiota characterization of *Exaiptasia diaphana* from the Great Barrier Reef

**DOI:** 10.1186/s42523-020-00029-5

**Published:** 2020-04-05

**Authors:** Leon Michael Hartman, Madeleine Josephine Henriette van Oppen, Linda Louise Blackall

**Affiliations:** 1 Swinburne University of Technology, Melbourne, Australia; 2 The University of Melbourne, Melbourne, Australia; 3 Australian Institute of Marine Science, Townsville, Australia

**Keywords:** Coral, *Aiptasia pallida*, *Exaiptasia pallida*, Model animal, Bacteria, *Symbiodiniaceae*

## Abstract

**Background:**

Coral reefs have sustained damage of increasing scale and frequency due to climate change, thereby intensifying the need to elucidate corals’ biological characteristics, including their thermal tolerance and microbial symbioses. The sea anemone, *Exaiptasia diaphana*, has proven an ideal coral model for many studies due to its close phylogenetic relationship and shared traits, such as symbiosis with algae of the family *Symbiodiniaceae*. However, established *E. diaphana* clonal lines are not available in Australia thus limiting the ability of Australian scientists to conduct research with this model. To help address this, the bacterial and *Symbiodiniaceae* associates of four Great Barrier Reef (GBR)-sourced *E. diaphana* genotypes established in laboratory aquaria and designated AIMS1–4, and from proxies of wild GBR *E. diaphana* were identified by metabarcoding of the bacterial 16S rRNA gene and eukaryotic rRNA gene ITS2 region. The relationship between AIMS1–4 and their bacterial associates was investigated, as was bacterial community phenotypic potential. Existing data from two existing anemone clonal lines, CC7 and H2, were included for comparison.

**Results:**

Overall, 2238 bacterial amplicon sequence variants (ASVs) were observed in the AIMS1–4 bacterial communities, which were dominated by *Proteobacteria* and *Bacteroidetes*, together comprising > 90% relative abundance. Although many low abundance bacterial taxa varied between the anemone genotypes, the AIMS1–4 communities did not differ significantly. A significant tank effect was identified, indicating an environmental effect on the microbial communities. Bacterial community richness was lower in all lab-maintained *E. diaphana* compared to the wild proxies, suggesting a reduction in bacterial diversity and community phenotypic potential due to culturing. Seventeen ASVs were common to every GBR lab-cultured anemone, however five were associated with the *Artemia* feedstock, making their specific association to *E. diaphana* uncertain. The dominant *Symbiodiniaceae* symbiont in all GBR anemones was *Breviolum minutum*.

**Conclusion:**

Despite differences in the presence and abundance of low abundance taxa, the bacterial communities of GBR-sourced lab-cultured *E. diaphana* are generally uniform and comparable to communities reported for other lab-cultured *E. diaphana*. The data presented here add to the global *E. diaphana* knowledge base and make an important contribution to the establishment of a GBR-sourced coral model organism.

## Background

Coral reefs are reservoirs of enormous biodiversity, are essential for the maintenance of marine and coastal ecosystems [1], and their economic and social values are vast [2]. Alarmingly, the loss of the coral’s energy-producing algal endosymbionts, a process known as bleaching, has increased in frequency and severity due to elevated sea surface temperatures (SST) caused by rising atmospheric greenhouse gas concentrations [3]. This has led to widespread coral mortality and damage to reef systems [4], and has heightened the need to investigate mitigation solutions. As reefs succumb to the impacts of climate change, the need for a model organism to assist coral research has never been greater. Fortunately, this need has been met in the form of the tropical sea anemone, *Exaiptasia diaphana* (previously *Aiptasia pallida* [5, 6]). The traits that make it an attractive candidate, particularly its intracellular symbiosis with the same algal family harbored by corals (*Symbiodiniaceae*) and lost under stress conditions, have seen it widely adopted by the research community, and several clonal lines have become established. However, none are available in Australia thus hampering the ability of Australian researchers to use this model for laboratory-based research and, consequently, a native Australian *E. diaphana* model is urgently needed.

*E. diaphana* CC7 and H2 are the clonal lines primarily employed in research. CC7 was developed from a single propagule of Atlantic Ocean origin obtained from Carolina Biological Supply (Burlington, North Carolina) [7]. H2 was developed from a founder collected at Coconut Island, Hawaii [8]. Distinguishing features are their gender and algal symbiont; CC7 is male and harbors *Symbiodiniaceae* of the genus *Symbiodinium*, whereas H2 is female and harbors *Breviolum* [9]. Baseline multi-omics data describing *E. diaphana* have been generated from these genotypes to elucidate cnidarian physiology [7, 10–12]. A key element of this work has been characterization of *E. diaphana*’s associated bacteria due to their influence on host health [13].

For example, Röthig et al. [14] investigated the bacterial associates of lab-reared CC7 by metabarcoding of the V5 to V6 region of the 16S rRNA genes. They compared bacteria associated with CC7 anemones either inoculated with *Breviolum minutum* or lacking *Symbiodiniaceae* (i.e., aposymbiotic), and the phenotypic potential inferred from the bacterial associates via the tool METAGENassist [15], as well as a core bacterial community. The number of bacterial OTUs per symbiotic anemone was modest (109 to 133) compared to some corals [16, 17]. Almost all OTUs belonged to the phyla *Proteobacteria* (67%), *Actinobacteria* (26%), *Bacteroidetes* (3%) or *Firmicutes* (2%). A core community i.e., OTUs present in every sample, of 37 OTUs was reported. However, the probability of an OTU being present in all samples was high because the samples originated from the same culture collection and few animals were compared (*n* = 5). The presence or absence of *Symbiodiniaceae* endosymbionts appeared to drive differences in inferred bacterial phenotype. For example, the bacterial communities of aposymbiotic anemones were depleted in sulfur metabolizing capacity. This was attributed to the absence of dimethylsulfoniopropionate (DMSP), which is generally produced by algal symbionts [18].

Brown et al. [19] described the bacteria of *E. diaphana* from the North Pacific, Atlantic Ocean and the Caribbean Sea using samples from museum collections, laboratory aquaria and pet shops. Metabarcoding of the V1-V4 of the 16S rRNA genes generated 12,585 operational taxonomic units (OTUs) from 49 samples. The anemones with the highest bacterial community richness were raised in artificial environments (1358–1671 OTUs), whereas those with the fewest were from Hawaii’s coastal waters (409 OTUs). The relative abundance of bacterial phyla varied greatly among the anemones but were consistent within environment types. For example, *Proteobacteria* (> 50%) dominated among wild Pacific, Atlantic and Caribbean anemones, whereas *Firmicutes* (~ 70%) dominated the bacteria of anemones from an outdoor laboratory aquarium. A core bacterial community was not found, but six genera (*Vibrio*, *Nautella*, *Ruegeria*, *Marinobacter*, *Lentisphaera* and *Flavobacterium*) were common. The authors concluded that *E. diaphana*’s bacterial communities are highly variable and shaped largely by their environment. However, the unusual sample origin and treatment of some (e.g., ethanol preserved museum specimens), disparate rearing conditions, and small sample sizes for each sample type (*n* ≤ 4) likely influenced these findings.

Herrera et al. [20] analyzed the bacterial microbiota of lab-reared H2 (*n* = 5) and CC7 (*n* = 5). Both genotypes were inoculated with *B. minutum* to standardize their algal symbionts. Bacterial community richness was lower in H2 than CC7 with 96 versus 118 OTUs, respectively. *Proteobacteria* was the most abundant phylum in both H2 (53%) and CC7 (70%), which matched the dominance of *Proteobacteria* in CC7 (67%) previously reported [14]. However, the relative abundance of *Bacteroidetes* (37%) and *Actinobacteria* (10%) in H2 differed markedly from CC7 (2% versus 26% respectively). Approximately 40% of OTUs were present in all five H2 samples. Once again, the common environment and small sample number increased the probability of identifying a core contingent. The phenotypic potential of each genotype’s bacterial associates was inferred via METAGENassist [15]. Due to their different compositions, the inferred phenotypes of H2 and CC7’s bacterial associates differed substantially. The most distinctive differences were in utilization of chitin, xylan, sugars and propionate, where H2 was depleted and CC7 enriched in each case. The different geographic origin and genders of the genotypes, rather than genotype itself, were offered as possible explanations for the distinct bacterial communities. This partly supported the previous conclusion [19] that environment shapes *E. diaphana*’s bacterial microbiota. However, it also assumed long-term stability of bacterial communities in culture, which has not been tested in *E. diaphana*.

The above reports provided the first insights into *E. diaphana*’s bacterial associates, but some aspects, such as richness estimates and the influence of host genotype, remain unclear. Information on *E. diaphana* sourced from the GBR is also absent. Here, we established cultures of GBR-sourced *E. diaphana*, and characterized their associated bacteria using a 16S rRNA gene metabarcoding approach. We incorporated data from the earlier studies [14, 19, 20] to provide a broad *E. diaphana* story. We explored genotypic influence on bacterial community composition and bacterial inferred phenotypes. Our establishment of a GBR-sourced *E. diaphana* model and characterization of its bacterial associates will help clarify *E. diaphana* associated microbiota variability and assist Australian coral researchers who have not had access to this rising star of coral research.

## Materials and methods

### *E. diaphana* culture collection

Anemones used in this study were taken from the *E. diaphana* culture collection at the University of Melbourne (UoM), Australia. This collection was established from late 2014 to early 2016 with anemones sourced from aquaria in the National Sea Simulator (SeaSim) at the Australian Institute of Marine Science (AIMS), Townsville, Australia, which are stocked with material from the GBR. The UoM collection contains four *E. diaphana* genotypes: AIMS1 (female), AIMS2 (male), AIMS3 (female) and AIMS4 (female) [21]. All are maintained at 26 °C under 12–20 μmol photons m^− 2^ s^− 1^ on a 12 h:12 h light-dark cycle and fed twice-weekly with freshly-hatched *Artemia salina*. Water is 100% changed once weekly with seawater reconstituted from Red Sea Salt™ at ~34 parts per thousand (See Additional file 1).

### Sampling and sample processing

Anemones were sampled in November 2017 for bacteria and *Symbiodiniaceae* characterization. Six anemones were randomly collected with 3 mL plastic sterile Pasteur pipettes from each of 12 culture tanks comprising three replicate tanks per genotype (Supplementary Figure S3, Additional file 1). Anemones were placed into sterile 1.5 mL centrifuge tubes, snap frozen in liquid nitrogen and stored at −80 °C until processing. One litre of water was siphoned from each tank and filtered through a 100 μm cell strainer (Falcon, 352360) into a sterile filter-unit (Nalgene, DS0320–5045), then through a 47 mm 0.2 μm membrane (Pall, 66234). The membranes were individually stored in sterile, covered Petri dishes (WestLab, 153–533) at −20 °C until processing. As the UoM *A. salina* feedstock was not presumed sterile, 3 × 75 μL of a dense *A. salina* suspension was sampled to identify associated bacteria.

*E. diaphana* is difficult to locate in the wild due to its cryptic nature, therefore five *E. diaphana* polyps were collected from the outflow of a 4000 L outdoor holding tank containing live corals, snails, sea cucumbers and fish at the AIMS SeaSim (See Supplementary Figure S4, Additional file 2). They were included to estimate the bacterial composition of GBR-sourced *E. diaphana* maintained in a complex marine environment and thus served as wild animal proxies. A 1 L water sample was also collected from the SeaSim holding tank and filtered through a 0.2 μm filter cartridge (Sterivex, SVGP01050), which was stored at −20 °C until processing (Table 1).

**Table 1:** Sampling summary for bacterial microbiota analysis

Sample type	Number of samples
*E. diaphana* (UoM AIMS1–4 cultures): 6 per tank per genotype (6 × 3 × 4)	72
Water (UoM AIMS1–4 cultures): 1 L per tank per genotype (1 × 3 × 4)	12
DNA extraction blanks: tissue × 1; water × 1	2
No-template PCR controls: × 3	3
*Artemia salina*: × 3	3
Wild proxy *E. diaphana* (from an AIMS SeaSim aquarium): × 5	5
Water (AIMS SeaSim aquarium): × 1	1
*E. diaphana* (CC7 16S rRNA gene sequencing data [14])	5
*E. diaphana* (H2 16S rRNA gene sequencing data [20])	5
Total	108

Sample DNA was extracted according to [22] but modified by 15 min incubation with 20 mL of 10 mg/mL lysozyme after sample homogenization, and 20 s bead beating at 30 Hz (Qiagen Tissue-Lyser II) with 100 mg of sterile glass beads (Sigma, G8772). For each water sample, filter membranes were sliced into thin strips with a sterile blade and treated as an animal tissue sample as previously described [14]. Blank extractions without sample material were used to test for reagent and plasticware contamination. Extracts were checked for DNA by agarose gel electrophoresis.

Bacterial DNA was amplified by PCR in triplicate using primers with Illumina adapters (not shown) targeting the V5-V6 regions of the 16S rRNA gene: 784F [5′ AGGATTAGATACCCTGGTA 3′], 1061R [5′ CRRCACGAGCTGACGAC 3′] [23], as previously used [14, 20]. Three no-template PCR blanks were included to test for reagent and plasticware contamination. To identify the anemones’ intracellular *Symbiodiniaceae*, DNA from 12 UoM *E. diaphana* (one from each tank) sampled for the bacterial analysis, and all five wild proxy anemone samples (Table 2),

**Table 2:** Sampling summary for *Symbiodiniaceae* analysis

Sample type	Number of samples
*E. diaphana* (UoM cultures): 1 per tank (1 × 12)	12
Wild proxy *E. diaphana* from SeaSim aquarium: × 5	5
Total	17

was amplified by PCR in triplicate using primers with Illumina overhangs (not shown) targeting the rRNA gene internal transcribed spacer region 2 (ITS2): ITS2-Dino-forward [5′ GTGAATTGCAGAACTCCGTG 3′] [24], ITS2rev2 [5′ CCTCCGCTTACTTATATGCTT 3′] [25]. Separate PCRs for bacteria and *Symbiodiniaceae* were performed in 20 μL volumes comprising 1 μL template DNA, 10 μL of 10 μM MyTaq HS Mix polymerase (Bioline), 0.5 μL of 10 μM forward primer, 0.5 μL of 10 μM reverse primer, and 8 μL MilliQ water. Thermal-cycler settings were: 1 cycle at 95.0 °C for 3 min, 30 cycles at 95.0 °C, 55.0 °C and 72.0 °C for 15 s each, and 1 cycle at 72 °C for 3 min. Triplicate PCR products were pooled and checked by agarose gel electrophoresis. The SeaSim water sample was removed from the analysis as no PCR product was visible on the agarose gels.

A volume of 25 μL of PCR product from each sample pool was sent to the Ramaciotti Centre for Genomics (RCG), Sydney, Australia for sequencing on a single Illumina MiSeq V2 (2 × 250 bp) run. RCG performed PCR product clean-up and normalization as part of sequencing library preparation.

### Sequencing data workflow

Raw, demultiplexed MiSeq reads were joined in QIIME2 v2018.4.0 [26]. Sequence denoising, chimera checking and trimming was performed in DADA2 [27] to correct sequencing errors, remove primer sequences and low quality bases. Resulting amplicon sequence variants (ASVs) with a single representative sequence were removed. Prokaryote taxonomy was assigned in QIIME2 against a SILVA database (v 132) trained with a naïve Bayes classifier [28–31]. ASVs identified as eukaryotes, mitochondria, or chloroplasts were removed. *Symbiodiniaceae* sequences, processed in DADA2 as above, were clustered into OTUs at 99% sequence similarity by closed-reference OTU picking in vsearch [32]. A database adapted from a study of *Symbiodiniaceae* diversity [33] was used for taxonomic classification and to seed the OTU clusters. The raw bacterial sequencing reads from the *E. diaphana* clonal genotypes CC7 and H2 previously reported [14, 20], respectively, were downloaded from NCBI’s Sequence Read Archive and processed as above for comparison.

All analyses described hereafter were performed in R v3.6.0 [34] with the packages vegan v2.5–6 [35], phyloseq v1.29.0 [36], microbiome v1.7.2 [37], mvabund v4.0.1 [38] ggplot2 v3.2.1 [39], DESeq2 v1.23.10 [40] and decontam v1.5.0 [41]. A significance threshold of α = 0.05 was used for all statistical tests, unless otherwise stated. Tabulated ASV counts, taxonomic assignments and metadata were imported into R, and rarefaction curves were generated to confirm that sequencing captured species diversity. Contaminant ASVs introduced during sample preparation were identified using the bacterial community data from the negative control samples using decontam’s ‘prevalence’ method and default threshold (*p* = 0.1). Stacked bar-charts of family-level ASVs were generated to assess sample bacterial community compositions.

### Diversity analyses

The bacterial metabarcoding data were normalized by subsampling to 12,810 sequences per sample. Bacterial community richness was assessed according to the number of observed ASVs per sample. Simpson [42] and Shannon index values [43] were used to describe and compare alpha diversity across the sample types. Differences in Shannon diversity between the sample types were evaluated by one-way analysis of variance (ANOVA) after checking for data normality and homogeneity of variance by Shapiro-Wilk [44] and Levene’s tests [45], respectively. Post hoc pair-wise comparisons were performed using Tukey’s HSD [46]. Relative proportions of bacterial phyla in all sample types were calculated and tabulated. Heatmaps of the 20 most abundant class-genus bacterial taxa in the AIMS1–4 and wild proxy anemones were generated and the magnitude (binary log fold change: L_2_FC) of significant pairwise differences were calculated to investigate whether differences in bacterial composition corresponded to sample type. nMDS ordinations (Hellinger transformation; Bray-Curtis dissimilarity) were generated of the AIMS1–4 samples to assess whether the genotypes’ bacterial communities were distinct. The genotype-bacteria relationship was explored using a Generalized Linear Model (GLM)-based approach with the data fitted to a negative binomial distribution and tested across 999 iterations. nMDS ordinations (Hellinger transformation; Bray-Curtis dissimilarity) of each AIMS1–4 genotype were plotted to investigate whether patterns indicative of non-random variation in the anemone-associated bacterial communities occurred within each genotype.

### AIMS1–4 core bacterial community member analysis

The AIMS1–4 bacterial communities were surveyed for core members. An ASV was deemed ‘core’ if it was present in every AIMS1–4 sample in accordance with the ‘shared membership’ criteria [47]. The core bacterial members in AIMS1–4 were also investigated in the water, wild proxy, CC7 and H2 anemones and the *A. salina* microbiota.

### Phenotypic potential analysis

The phenotypic potential of the sample’s bacterial associates was determined by the online tool METAGENassist [15], which maps taxonomy to phenotype using information from the BacMap [48], GOLD [49] and NCBI [50] databases. Data processing followed reported methods [14, 20]. Briefly, ASV count, taxonomic assignment, and sample type data were imported into METAGENassist. ASVs with identical taxonomic assignments were combined, and the data were filtered using interquartile range filtering to improve resolution and control the false discovery rate [51]. The remaining ASVs were normalized by sum for sample-to-sample comparison, and by Pareto scaling for taxon-to-taxon comparison. Data describing the phenotypic capability of each sample type in 15 categories previously assessed [14, 20] were exported from METAGENassist and displayed as a histogram.

## Results

### Bacteria metabarcoding

Sequencing produced 3,601,241 raw reads across AIMS1–4 and wild proxy *E. diaphana*, UoM water, *A. salina* feedstock and negative control samples: minimum 20,027; mean 37,126, maximum 60,798 reads per sample. After merging, denoising and chimera filtering, 2,516,454 reads remained: minimum 12,810, mean 25,943, maximum 44,033 reads per sample. A total of 4052 ASVs were identified. Incorporation of the CC7 and H2 data increased the number of ASVs in the dataset to 4587.

Rarefaction curves for bacterial sequences from all anemone and water samples plateaued, suggesting that sequencing depth was sufficient to capture bacterial species diversity (See Supplementary Figure S6, Additional file 3). Decontam [41] identified seven contaminant ASVs (0.06% of bacteria in the AIMS1–4 anemone samples, 0.07% in the AIMS1–4 water samples, and 2.08% in the wild proxies), which were removed (See Supplementary Table S1, Additional file 3). Three samples contained high relative abundances of ASVs from putatively contaminant bacterial taxa from the *Enterobacteriaceae* and *Vibrionaceae* (See Supplementary Figure S7, Additional file 3). These three samples were removed from further analyses, leaving 4401 ASVs across all sample types and 2238 ASVs associated with the AIMS1–4 anemones.

The wild proxy anemones contained on average, two and four times as many ASVs as the long-term lab-cultured AIMS1–4, and the CC7 and H2 *E. diaphana* genotypes, respectively (Table 3). Each AIMS1–4 water sample contained ~ 25% of the number of ASVs identified in the *E. diaphana* genotype grown in that water. *A. salina* had comparatively fewer bacterial associates, with only 27 ASVs.

**Table 3:** Number of bacterial ASVs in each sample type

Sample	Total ASVs
AIMS1 (*n* = 18)^a^	967 (205)^b^
AIMS 2 (*n* = 18)	860 (208)
AIMS 3 (*n* = 17)	810 (202)
AIMS 4 (*n* = 17)	758 (226)
Wild proxy *E. diaphana* from SeaSIM aquarium (*n* = 4)	1507
CC7 (*n* = 5)	439
H2 (*n* = 5)	317
*Artemia salina* (*n* = 3)	27

^a^Sample numbers after samples deemed to have been contaminated were removed. ^b^Number of bacterial ASVs in water samples in which the AIMS genotypes were raised are in parentheses

**Fig. 1:**
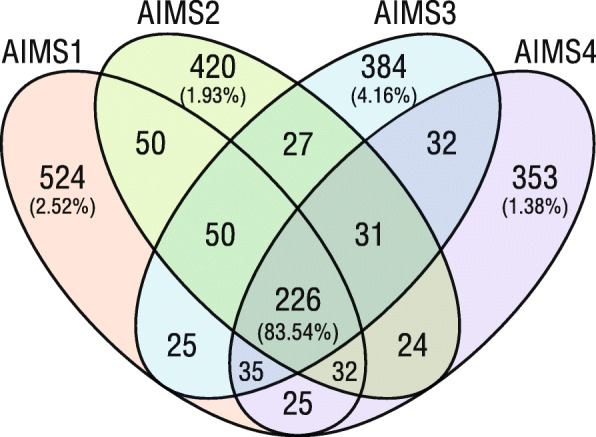
ASVs common to the AIMS1–4 genotypes. The total relative abundance of ASVs unique to each AIMS1–4 genotype are shown in parentheses

Although half the ASVs in each AIMS1–4 genotype were unique, they accounted for < 5% relative abundance of the communities (Fig. 1). However, ASVs common to AIMS1–4 averaged 83.54% total relative abundance, which suggested high similarity between the AIMS1–4 bacterial communities.

### Diversity analyses of anemone bacterial associates

**Fig. 2:**
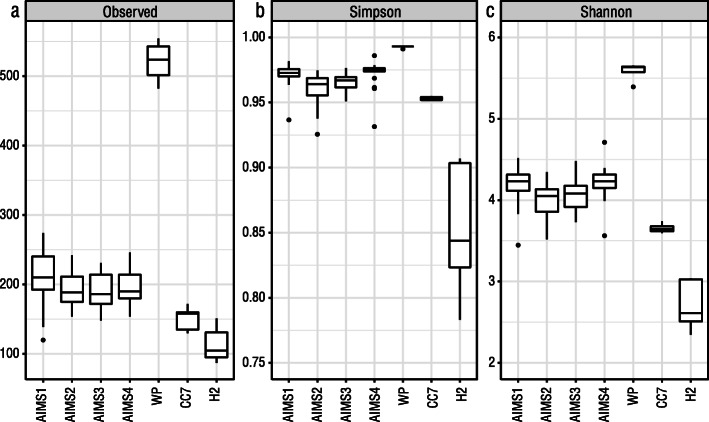
Alpha diversity of sample types. Alpha diversity was assessed by average number of observed ASVs – higher values indicate greater richness (**a**), Simpson diversity index – higher values indicate greater evenness (**b**), and Shannon diversity index – higher values indicate greater overall alpha diversity (**c**). WP = wild proxies

There was high variation in the number of bacterial ASVs observed within each sample type. The wild proxy anemones contained, on average, more than twice as many ASVs as the AIMS1–4 lab-cultured anemones (Fig. 2a; 521 versus 202). However, the Simpson values indicated that these two anemone groups had similar bacterial community evenness (Fig. 2b). The evenness of the H2 anemones was comparatively low (Fig. 2b), indicating dominance by a small number of ASVs. Collectively, the wild proxy anemones had considerably higher Shannon index values than the other anemones (Fig. 2c), demonstrating higher bacterial community diversity in wild proxies compared to lab-cultured anemones. The relatively low Shannon index value for H2 reflected its low observed ASV and Simpson index values.

Significant differences in alpha diversity, described by Shannon index, were detected between the sample types (ANOVA, *F*_6, 77_ = 65.19, *p* < 0.001). Subsequent pairwise testing by Tukey’s HSD indicated that the AIMS1–4 genotypes did not differ significantly from each other; however, the wild proxy anemones differed significantly from all other anemones due to their comparatively high bacterial richness and evenness. H2 also differed significantly from the other sample types but this was due to its low bacterial richness and evenness (see Supplementary Table S2, Additional file 4 for Tukey’s HSD results).

Proportions of bacterial phyla among the anemone sample types were compared with the AIMS1–4 samples pooled to provide a general overview of dominant phyla in the sample types. Eighteen phyla were identified in the AIMS1–4 samples; ~ 92% of the total bacterial community were members of *Proteobacteria* (~76%) or *Bacteroidetes* (~16%). *Spirochaetes* (2.54%) and *Acidobacteria* (1.83%) were the third and fifth most abundant bacterial phyla in AIMS1–4, but these phyla were considerably lower in the wild proxy, CC7 or H2 anemones (Table 4). In contrast, very low levels of *Actinobacteria* were detected in AIMS1–4 (0.58%) compared to the other anemones (Table 4). The wild proxy anemones were associated with 24 bacterial phyla. This was higher than in AIMS1–4 (18 phyla), CC7 (18 phyla) or H2 (10 phyla), which was not surprising given the considerably higher richness observed in the wild proxy anemones (Fig. 2a).

**Table 4:** Phyla identified in the collective AIMS1–4 animals, wild proxies, CC7 [14]^a^, H2 [20]^a^, and previously reported [19]. See Supplementary Table S3, Additional file 5 for individual AIMS1–4 values

Phylum	AIMS1–4 (%)	Wild proxies (%)	CC7 [14] (%)	H2 [20] (%)	Reported in Brown et al. (2017) [19]
*Proteobacteria*	76.21	64.59	67.18	52.94	✓^b^
*Bacteroidetes*	15.58	25.7	2.73	37.11	✓
*Spirochaetes*	2.53	0.17	0.02		✓
*Planctomycetes*	1.89	3.13	0.09	0.04	✓
*Acidobacteria*	1.83	1.22	0.01		✓
*Chlamydiae*	1.18	0.63	1.18	0.19	
*Actinobacteria*	0.58	3.31	26.27	9.33	✓
*Firmicutes*	0.14	0.16	2.28	0.26	✓
*Calditrichaeota*	0.02	0.03			
*Verrucomicrobia*	0.01	0.12			✓
*Cyanobacteria*	0.01	0.08			✓
*Gemmatimonadetes*	0.01	0.35			✓
*Dependentiae*	< 0.01	0.07		0.10	
*Patescibacteria*	< 0.01	0.08	0.02	< 0.01	
*WPS*-2	< 0.01				
*Elusimicrobia*	< 0.01		0.04		
*Lentisphaerae*	< 0.01			< 0.01	✓
*Fusobacteria*	< 0.01	0.04	0.03	0.01	✓
*Marinimicrobia* (SAR406 clade)			0.03		
*Deferribacteres*			0.01		
*Tenericutes*		0.04			✓
*Chloroflexi*		0.05			✓
*Kiritimatiellaeota*		0.11			
*Nitrospirae*		0.01	0.01		✓
*Armatimonadetes*		0.03	0.05		✓
*Latescibacteria*		0.01			
*PAUC*34*f*		0.01			
*Omnitrophicaeota*			0.03		
*Fibrobacteres*		0.04	0.02		✓
*Epsilonbacteraeota*		0.01	< 0.01	< 0.01	

^a^Values for CC7 and H2 may differ from those previously reported in [14, 20] due to differences in bioinformatic methods. ^b^ ✓ = detected; empty cell = not detected

**Fig. 3:**
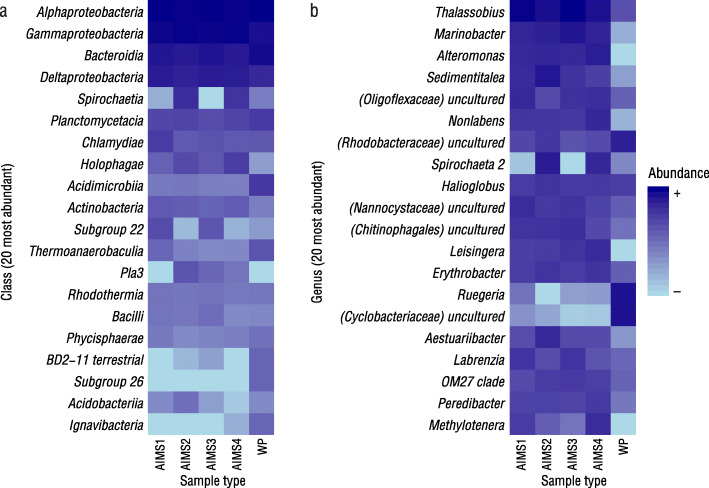
Heatmaps of the top 20 taxa by relative abundance at class (**a**), and genus levels (**b**)

Bacterial community composition was clearly different between the AIMS1–4 and wild proxy anemones at both the class level (Fig. 3a) and the genus level (Fig. 3b). For example, whilst the phylum level data showed a high relative abundance of *Spirochaetes* in AIMS1–4 (2.53%) (Table 4), as seen in the class-level heatmap, *Spirochaetia* occurred almost exclusively in AIMS2 and AIMS4. Analysis by DESeq2 confirmed that the L_2_FC difference in *Spirochaetia* between AIMS2 and AIMS4 and all other sample types was significant (See Supplementary Table S4, Additional file 6). Further, members of Subgroup 22 (phylum *Acidobacteria*) occurred in AIMS1 and AIMS3 but were rare in other anemones, and *Pla3* bacteria (phylum *Planctomycetes*) were absent from AIMS1 and wild proxies but present in AIMS2–4. The magnitude of these differences (L_2_FC) were also significant. At the genus-level (Fig. 3b), *Alteromonas* was highly abundant in AIMS1–4 but absent in the wild proxy anemones, and *Ruegeria* was highly abundant in the wild proxy anemones but present only at low levels in AIMS1–4. Differences in *Spirochaeta2* abundance followed the pattern observed for *Spirochaetia.* These differences (L_2_FC) were significant. See Additional file 6, for order and family-level heatmaps and all L_2_FC data.

**Fig. 4:**
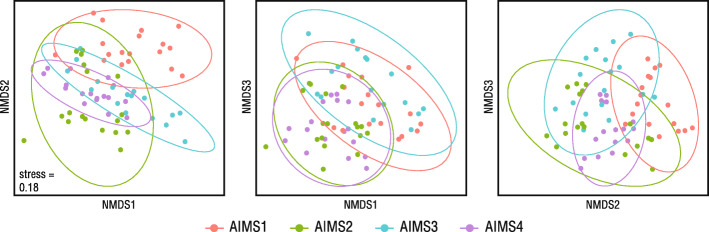
Comparison of bacterial communities in Ep1–4 anemone samples by nMDS on Bray-Curtis distances. All projections of 3D nMDS ordination are shown. Ellipses indicate 95% confidence intervals

Although the heatmaps suggested the existence of a genotype-specific bacterial association in AIMS1–4, an nMDS ordination showed little separation by genotype (Fig. 4).

**Fig. 5:**
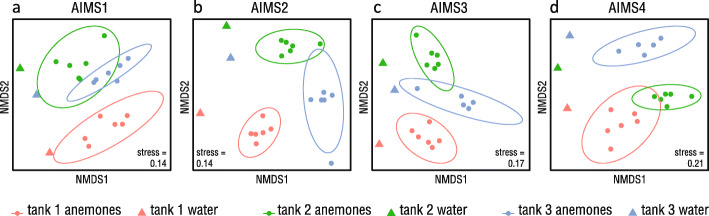
Comparison of bacterial communities in water and anemones by nMDS on Bray-Curtis distances. AIMS1 (**a**), AIMS2 (**b**), AIMS3 (**c**), and AIMS4 (**d**). Ellipses indicate 95% confidence intervals

The similarity of the AIMS1–4 bacterial communities was also demonstrated in testing by GLMs. Due to a significant interaction between genotype and tank (LRT = 3506, *p* < 0.001) (see Supplementary Table S8, Additional file 7) these variables were assessed separately. Testing revealed that whilst, overall, there was no significant difference in community composition based on anemone genotype (LRT = 0, *p* = 0.997), there was a significant tank-wise difference between the samples (LRT = 5941, *p* < 0.001) (see Supplementary Table S9, Additional file 7). This suggested the presence of a tank effect. Accordingly, nMDS ordinations of the data split by genotype showed general tank-wise sample separation (Fig. 5). The proximity of water and anemone datapoints also indicated a consistent relationship between waterborne and anemone bacteria.

#### Symbiodiniaceae

Sequencing to identify *Symbiodiniaceae* produced 339,727 raw reads across the 12 representative AIMS1–4 samples and the five wild proxy anemones (minimum 12,097; mean 19,984, maximum 34,839). After merging, denoising and chimera filtering, 320,108 reads remained (minimum 11,092, mean 18,830, maximum 33,461). At a 99% sequence similarity clustering threshold, 307,402 reads formed a single OTU identified as *Breviolum minutum* (previously *Symbiodinium* Clade B, sub-clade B1) which was present in all AIMS1–4 lab-cultured and wild proxy anemones. Two as-yet unnamed *Breviolum* OTUs (previously *Symbiodinium* sub-clades B1i and B1L) were also identified, with each containing 697 and 445 reads, respectively. B1i was identified in 16 samples, whereas B1L was identified in one. In a single wild proxy anemone, two OTUs of seven reads each were assigned to the *Symbiodiniaceae* genera *Cladocopium* (previously *Symbiodinium* Clade C) and *Durusdinium* (previously *Symbiodinium* Clade D) [52]. The remaining 11,550 reads were unassigned.

### AIMS1–4 core bacteria

Seventeen AIMS1–4 core ASVs were identified (Table 5). All were also detected in the water samples, with one *Saprospiraceae* ASV being particularly prevalent. No AIMS1–4 core ASVs were identified in the wild proxy anemones, and only two and five AIMS1–4 core ASVs were present in the CC7 and H2 anemones, respectively. Five AIMS1–4 core ASVs were also detected in the *A. salina* feedstock. Three core ASVs of the genus *Thalassobius* were particularly abundant in the AIMS1–4 anemones, collectively accounting for 8.78% relative abundance in those samples. A core *Alteromonas* ASV was present in high abundance in all but the wild proxy samples and comprised 51.84% of the *A. salina* bacterial community. In contrast, a core *Sedimentitalea* ASV was common to all sample types except *A. salina*. No core ASVs were identified to species level during bioinformatic processing (see Supplementary Table S10, Additional file 8 for taxonomic naming and ASV sequences).

**Table 5:** Relative abundance of bacterial ASVs^a^ present in every AIMS1–4 sample, and co-occurrence in other samples^b^ across all samples within each sample type

	*Phylum – Family; Genus*	AIMS 1–4 (%)	AIMS 1–4 water (%)	Wild proxies (%)	CC7 (%)	H2 (%)	*Artemia salina* (%)
1	*Proteobacteria – Rhodobacteraceae; Thalassobius*	5.09	0.31				
2	*Bacteroidetes – Saprospiraceae*	3.98	20.18				
3	*Proteobacteria – Rhodobacteraceae; Sedimentitalea*	3.59	0.78	0.06	0.76	3.80	
4	*Proteobacteria – Alteromonadaceae; Alteromonas*	3.30	1.28		2.44	2.16	51.84
5	*Proteobacteria – Alteromonadaceae; Marinobacter*	2.74	0.80				0.62
6	*Proteobacteria – Rhodobacteraceae; Thalassobius*	2.65	0.15				
7	*Proteobacteria – Oligoflexaceae*	2.01	0.10	0.06		0.59	
8	*Proteobacteria – Rhodobacteraceae; Leisingera*	1.93	0.18		0.18		0.38
9	*Proteobacteria – Alteromonadaceae; Marinobacter*	1.93	4.59				
10	*Planctomycetes – Rubinisphaeraceae*	1.42	0.10				
11	*Proteobacteria – Hyphomonadaceae*	1.22	2.23	0.66		0.60	
12	*Proteobacteria – Bacteriovoracaceae; Peredibacter*	1.14	0.62		0.01		
13	*Proteobacteria – Pseudohongiellaceae; Pseudohongiella*	1.12	3.63				
14	*Proteobacteria – Rhodobacteraceae; Thalassobius*	1.04	1.71				8.73
15	*Proteobacteria –* (Order: *Cellvibrionales*)	0.75	0.61				
16	*Proteobacteria – Alcanivoracaceae; Alcanivorax*	0.45	0.30			0.01	0.01
17	*Proteobacteria – Bdellovibrionaceae; Bdellovibrio*	0.25	0.38	0.01			

^a^ASVs are described to the deepest taxonomic level identified during bioinformatic processing. ^b^Empty cell = not detected within sample type

### Phenotypic potential analysis

**Fig. 6:**
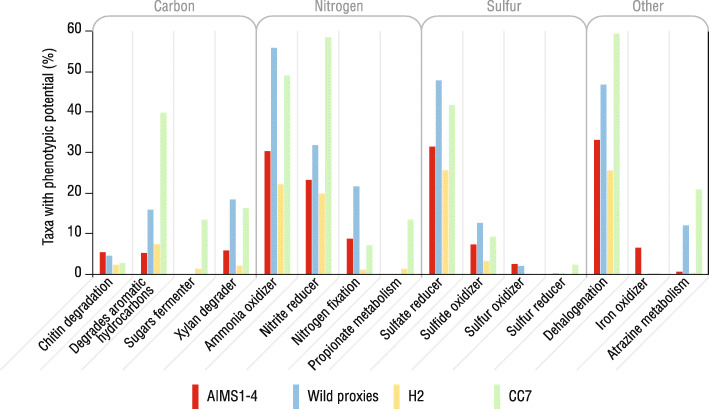
Putative phenotypic potential of bacteria in each *E. diaphana* sample type. Vertical bars indicate the percentage of bacterial taxa in each sample type with the phenotypic potential for each metabolism category according to taxonomy-to-phenotype mapping

The phenotypic potential of bacteria associated with the AIMS1–4, wild proxy, CC7 and H2 anemones was determined in METAGENassist [15]. After processing, 99 metabolism variables were retained and all anemones were compared across 15 metabolism categories previously described [14, 20] (Fig. 6). There was high variability between the sample types, but the inferred nitrogen and sulfur metabolism, and dehalogenation potential of their bacterial taxa was consistently high compared to the other categories. AIMS1–4 bacteria were enriched in iron oxidation capability, and depleted in sugar fermentation, and propionate and atrazine metabolism, compared to other anemones. The phenotypic potential of the AIMS1–4 samples was generally depleted compared to the wild proxy anemones.

## Discussion

### Bacterial associates of the anemones

In this study of GBR-sourced *E. diaphana*, lab-cultured anemones, AIMS1–4, were associated with considerably fewer ASVs than wild proxy anemones. The lab-cultured anemones were maintained for several years in semi-sterile sea water (Red Sea Salt prepared with RO water) and fed commercial *A. salina* with few bacterial associates, whereas the wild proxies were from an environment containing a large variety of marine animals likely resulting in exposure to diverse bacteria. Therefore, we conclude that the culture environment (lab-maintained versus AIMS aquarium) explains the bacterial community differences.

Differences in alpha diversity between the AIMS1–4 and wild proxy bacterial communities illustrated a key difference between lab-cultured and non-cultured anemones. Low alpha diversity of the AIMS1–4 bacteria compared to the wild proxies suggested a reduction in bacterial community complexity over time in culture. CC7 and H2 have been cultured for at least ten [7] and seven [8] years, respectively, and had the least diverse bacterial associates, which supports this hypothesis. The suggestion of a shift towards bacterial simplicity in culture is also supported by a study in which the diversity of bacteria associated with *E. diaphana* transferred from aquaria containing complex species to a laboratory environment dropped from 884 ± 104 OTUs to 523 ± 209 OTUs after 4 months [19]. Such a reduction in the diversity of anemone-associated bacteria may be due to the simple, stable nature of the culturing system. Alternatively, lab-culturing may have reduced bacterial diversity to a ‘minimal microbiome’, or to “the smallest set of microbes and/or microbial functions needed to develop a stable community” [53].

Bacterial communities in the AIMS1–4 tank water were simple compared to the resident anemones, suggesting that conditions (e.g., nutrient levels, pH) compared to those in and around the anemones (e.g., in the SML and gastrovascular cavity) supported lower bacterial diversity. It could also be due to some bacterial members being strict anemone symbionts, or dilution due to the regular full water changes, which meant bacterial seeding to the water was only from the anemones and air. Since the SeaSim water sample generated no PCR product, we could not compare the bacteria of the wild proxy anemones and their environment.

The bacterial communities of AIMS1–4 were dominated by five phyla, particularly *Proteobacteria* and *Bacteroidetes*. These two phyla also dominated the bacterial communities of the wild proxy anemones and are common in corals [54], which is relevant given the use of *E. diaphana* as a coral model. The third most prevalent AIMS1–4 bacterial phylum, *Spirochaetes*, was unusual in its high relative abundance compared to the other anemones. However, this was only found in AIMS2 and AIMS4 whose culture histories differed from AIMS1 and AIMS3 (refer Additional file 1). *Spirochaetes* has been reported in high abundance in some coral species [55], but their role was not elucidated.

Although some patterns of bacterial community composition among the AIMS1–4 anemones appeared to be genotype-related, the microbiota of AIMS1–4 were not found to be significantly different overall. It is possible however, that genotype-driven differences did exist but were obscured by variability caused by a tank effect as a similar, albeit non-significant, effect was observed in a recent study comparing the bacterial associates of *E. diaphana* maintained at different temperatures [56].

The influence of species versus environment in shaping coral-associated microbiota has been assessed in more than 60 studies, with an almost even split between findings of either species-specific or spatio-temporal-driven association [57]. The data from the present study suggest that *E. diaphana* and its bacterial members fall into the second category. However, the aforementioned binary assignment oversimplifies the nature of cnidarian bacterial communities, which are renowned for their complexity [58]. For example, coral microbiota vary depending on host compartment (e.g. gastrovascular cavity versus SML) and life-stage [59, 60]. Subsequently, studies that sample *E. diaphana* of different ages and from different compartments are needed to clarify the host-bacterial relationship.

#### *Symbiodiniaceae*

Almost all *Symbiodiniaceae* associated with the AIMS1–4 anemones were identified as *Breviolum minutum*. This high level of host-symbiont specificity was consistent with previous findings that Pacific Ocean *E. diaphana* associate exclusively with *B. minutum* [61]. Despite this, the detection of two other *Breviolum* species, and *Symbiodiniaceae* from *Cladocopium* and *Durusdinium* in the GBR-origin anemones may suggest that GBR *E. diaphana* live in symbioses with a mix of *Symbiodiniaceae* types. However, the *Cladocopium* and *Durusdinium* OTUs contained few reads and were found in a single wild proxy anemone, and therefore may have been planktonic *Symbiodiniaceae* that were sampling bycatch.

### *E. diaphana* core bacteria

There were few core ASVs among the AIMS1–4 anemones compared to the other lab-reared anemones, CC7 and H2, or the wild proxies. However, the small number of samples in the original CC7 and H2 studies increased the probability of ASVs being common to those samples. The absence of the AIMS1–4 core ASVs in the wild proxy anemones reiterated the difference between bacterial associates of lab-cultured *E. diaphana* versus those from a more complex environment. The presence of all core ASVs in the AIMS1–4 water samples likely indicates that the culture environment favored their growth and hence their ubiquitous association with *E. diaphana*. For example, some members of *Saprospiraceae*, which was highly abundant in the water samples, are ‘defining members of biofilms’ on plastics in the marine environment [62] so may have found the culture tank walls an ideal growth substrate [63].

Core bacteria may include those detectable and important to holobiont function [64]. They may also include ‘conditionally rare taxa’ that are present at levels below detection that proliferate under favorable conditions [47], or taxa that are introduced and become established. The presence of an *Alteromonas* core ASV in AIMS1–4, CC7 and H2, but absence from the wild proxy anemones, suggests proliferation or introduction, rather than functional importance. For example, the *Alteromonas* bacterium, *A. macleodii*, is widely distributed in the marine environment and is an r-strategist, i.e., it opportunistically blooms when nutrients are in high concentration [65]. Therefore, the abundance and ubiquity of the core *Alteromonas* ASV could be due to high nutrient levels in the culturing systems. Alternatively, it may have been introduced via the *A. salina* feedstock that had bacterial communities comprising > 50% of the *Alteromonas* core ASV and was fed regularly to the AIMS1–4 anemones. This may also apply to the *Marinobacter* and *Thalassobius* core ASVs, and due to their correspondence with *A. salina* we cannot assume they are important players in the *E. diaphana* holobiont.

Two core *Thalassobius* ASVs comprised ~8%G of the collective AIMS1–4 bacterial communities. However, they were not detected in the other anemones and thus did not match the *Thalassobius* ASV previously reported as a core member in CC7 and H2 [14, 20]. *Thalassobius* have been identified in many coral studies, particularly studies on coral bleaching [66] and diseases [67, 68], but no specific role, pathogenic or otherwise, was suggested. However, according to genomic analysis some strains degrade dimethylsulfoniopropionate (DMSP) [69], which is produced by *Symbiodiniaceae* [70] and coral [18], but not *E. diaphana* [71]. Therefore, the core *Thalassobius* ASVs may be involved in sulfur cycling.

### Phenotypic potential of anemone-associated microbiota corresponds to culture environment

The phenotypic potential of the AIMS1–4 bacterial communities was generally depleted compared to the wild proxy anemones, which may be due to their lower diversity. However, chitin degradation, which might provide carbon for metabolism, was marginally higher in AIMS1–4 than the wild proxies. Carbon is acquired by coral primarily from its intracellular algal symbiont as excess photosynthate or through heterotrophy [72] but resident bacteria are also important in carbon cycling [73], including through chitin-degradation [74]. Therefore, bacteria may cycle carbon in the *E. diaphana* holobiont by metabolising chitin, such as from *A. salina* exoskeletons, as a food source [75]. In contrast, xylan-degradation was somewhat higher in the wild proxy anemones than AIMS1–4. Xylan is a cell wall component in many green algae [76], including those found in coral reef systems [77]. Thus, xylan-degrading bacterial associates of AIMS1–4 may degrade green algae that are pest species in the culturing system.

Research has suggested that corals can assimilate nitrogen directly through uptake and processing of dissolved ammonium [78]. However, since most reefs exist in nitrogen-limited ecosystems, the contribution of nitrogen-fixing bacteria to coral holobiont function is critical [79, 80]. Therefore, it was not surprising that high nitrogen-processing potential was reported for the bacterial communities of all sample types, or that known coral-associated nitrogen-processing bacteria were identified in them, including *Cyanobacteria* [81] and *Rhizobiales* [57] (nitrogen fixation), and *Planctomycetes* [82].

Sulfur cycling is another important service provided to the host by coral-associated bacteria, and sulfate reducing potential registered highly in bacteria from all anemones. DMSP production by *Symbiodiniaceae* and processing by bacteria is known to be central to holobiont sulfur cycling and host acquisition [70, 83], and bacteria of the genera *Roseobacter*, *Vibrio*, and *Alteromonas* are capable of degrading DMSP to make sulfur available [83]. Taxa from each genus were present in the AIMS1–4 and wild proxy anemones, particularly *Alteromonas*, which was the third most abundant genus across the AIMS1–4 anemones.

Iron oxidation was the only category in which AIMS1–4 possessed higher phenotypic potential than the other anemones. Iron-oxidizing bacteria generally belong to the phylum *Proteobacteria*, and class *Zetaproteobacteria* [84]. Although the majority of taxa in AIMS1–4 were members of *Proteobacteria*, none were *Zetaproteobacteria*. However, some *Gammaproteobacteria* of the genus *Marinobacter* also oxidize iron [85]. This was one of the most abundant genera in AIMS1–4 compared, for example, to the wild proxy anemones, which may explain the relatively high iron-oxidizing potential for the AIMS1–4 bacterial associates.

Atrazine metabolism by the AIMS1–4 and wild proxy anemones was an interesting feature identified. Atrazine is an herbicide used extensively in the Queensland sugar cane industry that finds its way into the GBR via terrestrial run-off where it poses a risk to coral through its impact on *Symbiodiniaceae* [86–88]. Therefore, the ability of resident bacteria to degrade atrazine would be highly beneficial for anemones and corals on the GBR. Despite this, it is important to acknowledge that inferring function from taxonomy does not account for genes that may be present but not active. Future studies based on multi-omics analyses are required to distinguish the genomic potential and metabolic activity of *E. diaphana*’s bacterial associates.

## Conclusion

The GBR-sourced, lab-cultured *E. diaphana* in this study were generally consistent with previous model and wild proxy *E. diaphana* in terms of dominant bacterial associates, and resident *Symbiodiniaceae*. Bacterial richness was similar to other model *E. diaphana* samples but lower than wild proxy anemones, suggesting a loss of bacterial diversity in culture. The impact of this on *E. diaphana* health is unknown but could reduce holobiont phenotypic capability. Whilst there were differences in the bacterial associates hosted by different anemone genotypes (AIMS1–4), community-level differences were not statistically significant although these may have been obscured by tank-tank variation among the samples. Nevertheless, compositional differences provided an indication of *E. diaphana* bacterial community shifts due to environment, and for membership flexibility, which was further evidenced by the small core bacterial composition. By establishing GBR-sourced *E. diaphana* in lab-culture and producing baseline bacterial associate data we have laid the foundation for future laboratory-based research with this model organism in Australia and elsewhere, particularly if resident bacterial communities and their influence on holobiont function and resilience is of interest.

## Supplementary information

**Additional file 1: Figure S1.** Anemones acquired from the AIMS SeaSim in late 2014. **Figure S2.** Anemones acquired from the AIMS SeaSim in early 2016. **Figure S3.***E. diaphana* culture collection at the UoM.

**Additional file 2: Figure S4.** Holding tank at AIMS. **Figure S5.** Holding tank outflow with anemones.

**Additional file 3: Figure S6.** Rarefaction curves for all samples. **Figure S7.** Relative abundance of family level taxa. **Figure S8.** Legend of family-level taxa.

**Additional file 4: Table S2.** Tukey’s HSD *p*-values from pair-wise Shannon value comparison.

**Additional file 5: Table S3.** Relative abundance of phyla in each AIMS1–4 genotype.

**Additional file 6: Table S4.** Pairwise log_2_ fold change for 20 most abundant phylum-level taxa. **Table S5.** Pairwise log_2_ fold change for 20 most abundant genus-level taxa. **Figure S9.** Heatmaps of the top 20 taxa by relative abundance at order (a) and family (b) levels. **Table S6.** Pairwise log_2_ fold change for 20 most abundant order-level taxa. **Table S7.** Pairwise log_2_ fold change for 20 most abundant family-level taxa.

**Additional file 7: Table S8.** Output from a GLM-based analysis comparing the AIMS1–4 bacterial community compositions (‘tank’ nested within ‘genotype’). **Table S9.** Output from a GLM-based analysis comparing the AIMS1–4 bacterial community compositions (‘tank’ and ‘genotype’ as separate main effects).

**Additional file 8: Table S10.** Taxonomic naming and sequence data of the core AIMS1–4 ASVs.

## Data Availability

Illumina MiSeq data are available under NCBI BioProject PRJNA575811.

## Abbreviations

AIMS: Australian Institute of Marine Science
ASV: Amplicon Sequence Variant
DMSP: Dimethylsulfoniopropionate
GBR: Great Barrier Reef
GLM: Generalized Linear Model
ITS2: Internal Transcribed Spacer 2
L_2_FC: Log2 Fold Change (binary log fold change)
LRT: Likelihood-Ratio Test
nMDS: Non-metric Multidimensional Scaling
OTU: Operational Taxonomic Unit
rRNA: Ribosomal Ribonucleic Acid
SML: Surface Mucus Layer
SST: Sea Surface Temperature
UoM: University of Melbourne
