# Supplementary material for: Microbiota characterization of Exaiptasia diaphana from the Great Barrier Reef
Source: Anim Microbiome. 2020 Apr 5;2:10. doi: 10.1186/s42523-020-00029-5 (PMC7807684; doi:10.1186/s42523-020-00029-5)
Supplement: Supplementary file 1 — Additional file 1: Figure S1. Anemones acquired from the AIMS SeaSim in late 2014. Figure S2. Anemones acquired from the AIMS SeaSim in early 2016. Figure S3.E. diaphana culture collection at the UoM. [file 42523_2020_29_MOESM1_ESM.docx]

**Additional File 1**

Culture collection establishment

The *Exaiptasia diaphana* cultures in this study were taken from the culture collection at the University of Melbourne (UoM), Australia. Creation and maintenance of the collection, and details of species verification, genotyping and gender determination are described in [1]. However, for the present study, it should be noted that the anemones in the collection were acquired in two stages, and therefore have different culture histories. An initial group of *E. diaphana* were supplied to Swinburne University of Technology (SUT) in Melbourne, Victoria in late 2014 when coral skeleton fragments with attached anemones (Figure S1) were obtained from aquaria in the National Sea Simulator (SeaSim) at the Australian Institute of Marine Science (AIMS). These anemones were found to comprise two genotypes and genders, subsequently designated AIMS2 (male) and AIMS4 (female).


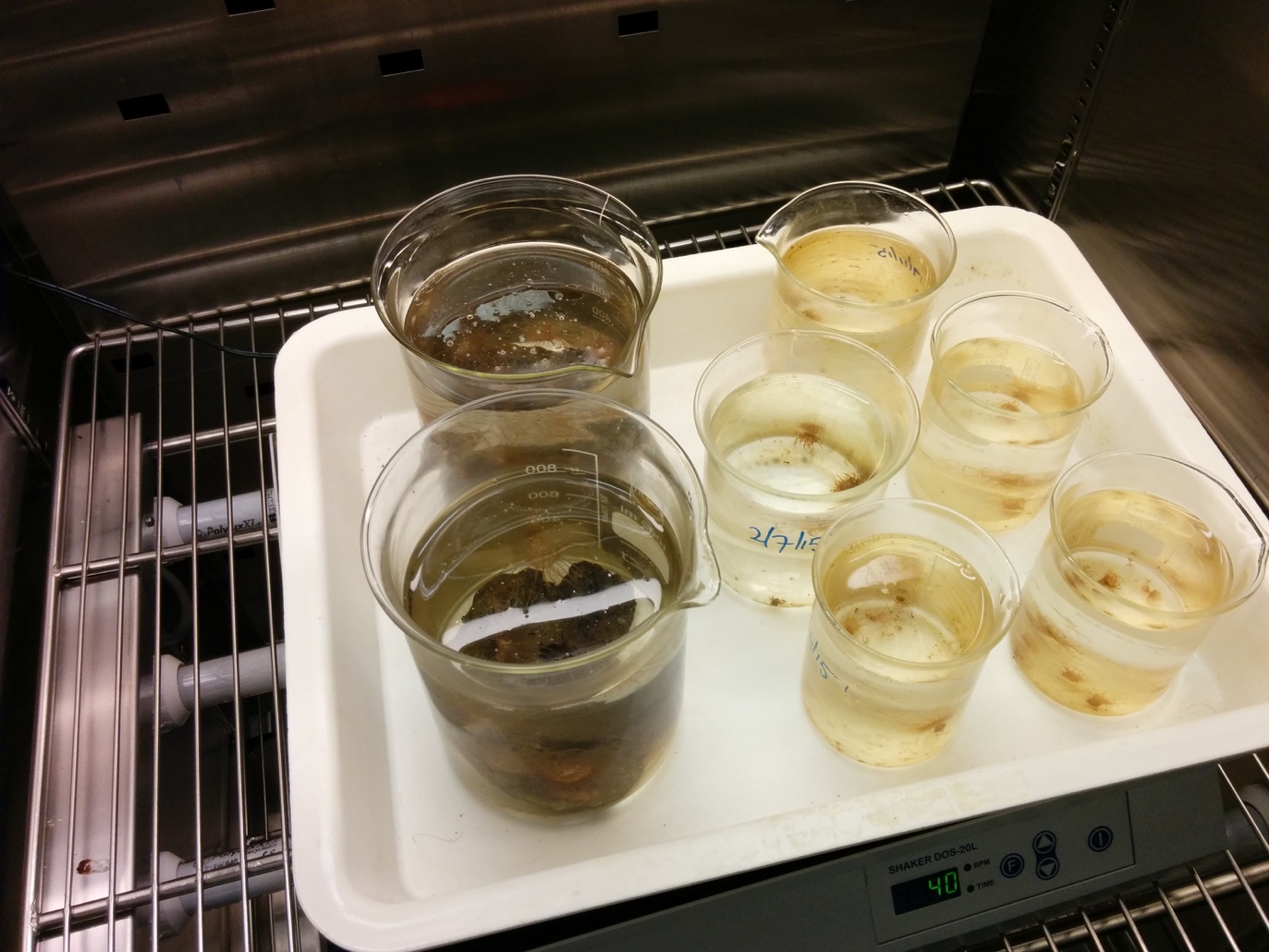


Figure S1: E. diaphana acquired from the AIMS SeaSim in late 2014. Original founder anemones are shown in two large beakers, and asexually generated offspring are shown in small beakers as at February 2016. Aeration was provided by orbital agitation at low speed.

In early 2016 a group of *E. diaphana* were supplied by AIMS to UoM, as above, and kept in a 6 L glass tank (Figure S2). These anemones were found to comprise two further genotypes and one gender, subsequently designated AIMS1 (female) and AIMS3 (female). In March 2017, all SUT anemones were transferred to UoM to create a single culture collection.


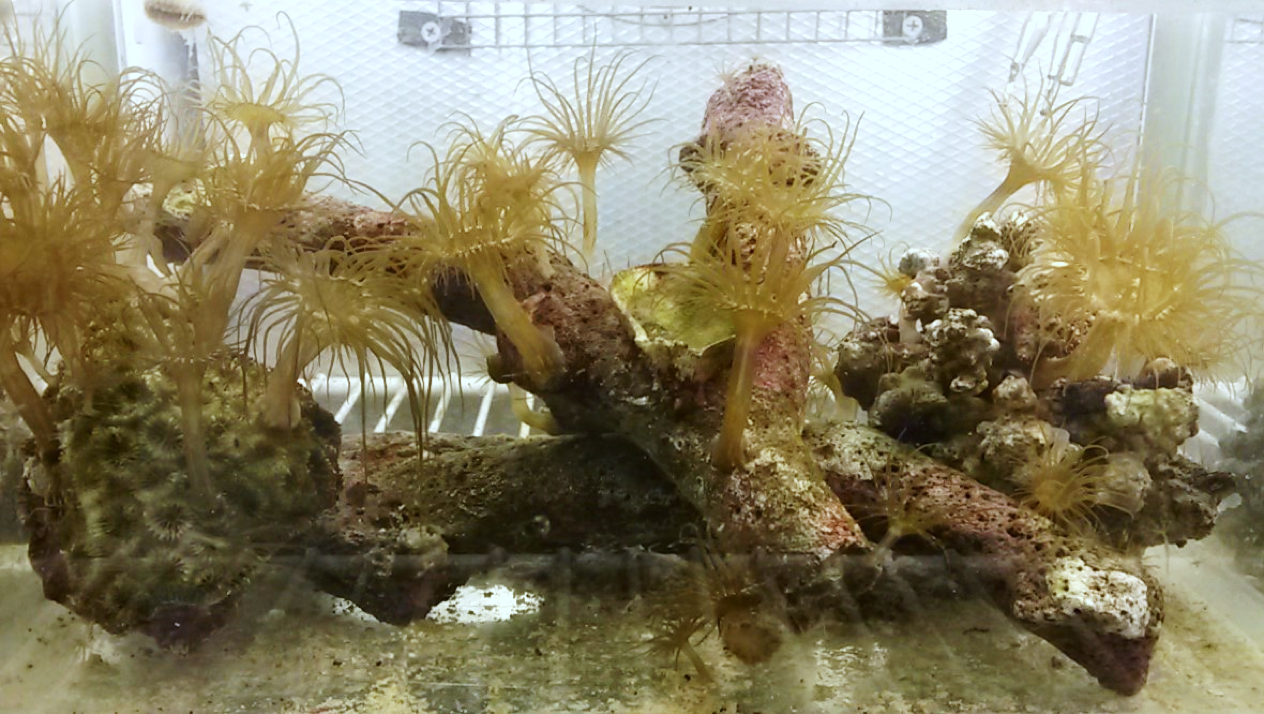


Figure S2: E. diaphana acquired from the AIMS SeaSim in early 2016. Anemones are shown as at October 2016. Aeration was provided by bubbling unfiltered air from a small aquarium pump.

Culture collection maintenance

The culture collection is maintained in a temperature-controlled walk-in incubator in 6 L clear polycarbonate tanks, with three tanks assigned per genotype to create three independent populations per genotype (Figure S1). The tanks are rotated between the shelves each week. Lighting is provided by white light LEDs strips at 12-20 µmol photons m^–2^ s^–1^ on a 12h:12h light-dark cycle. Temperature is maintained at 26 °C. The water is 100% changed once weekly with reconstituted seawater made from Red Sea Salt™ at ~34 parts per thousand. Feeding is performed twice weekly with freshly hatched *Artemia* nauplii. At the time of sampling for the present study, aeration was provided by bubbling unfiltered air from small aquarium pumps through air-stones.


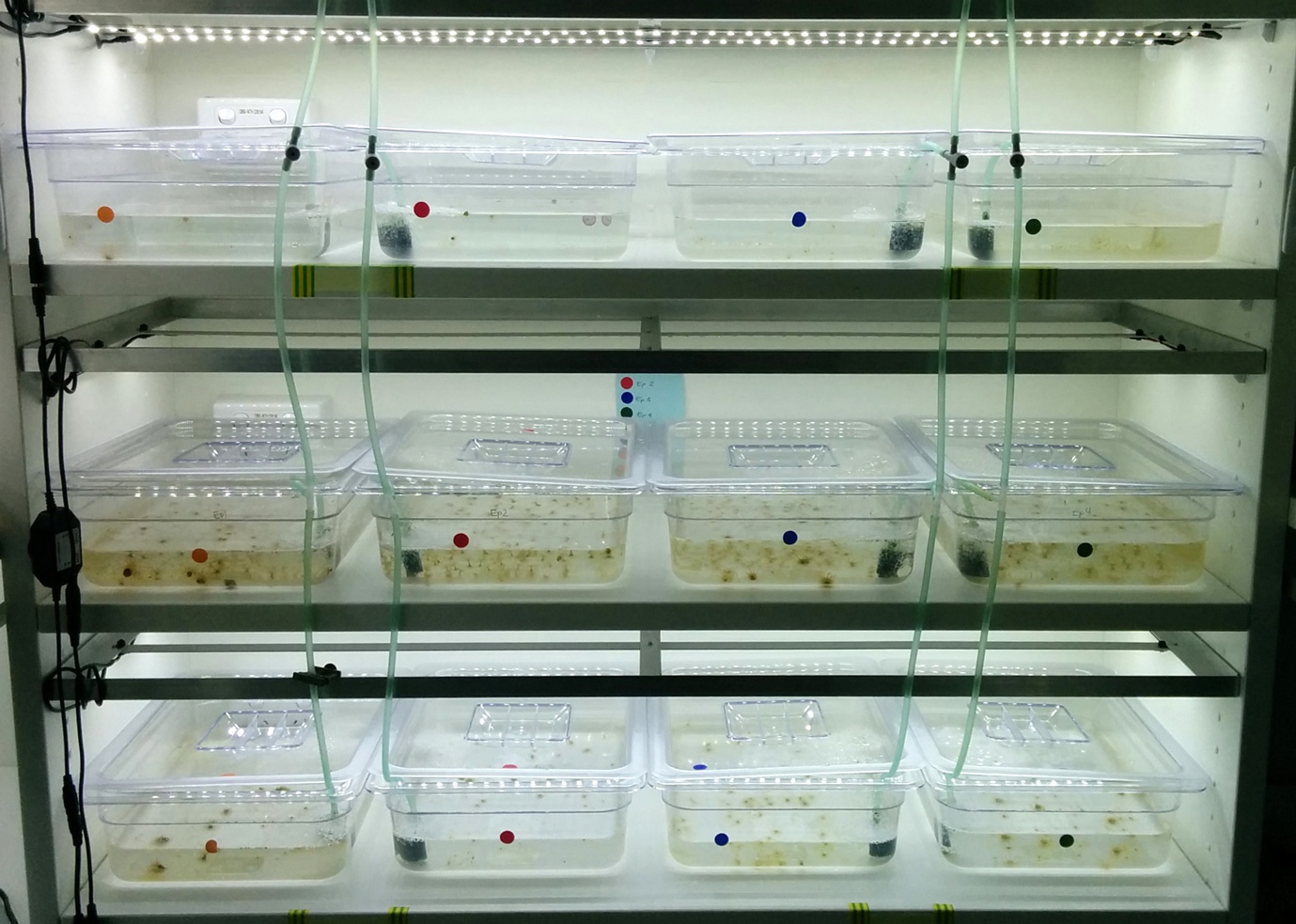


Figure S3: E. diaphana culture collection at the UoM. Genotypes AIMS1-4 are located left to right, with three replicate tanks located top to bottom. For the present study, six whole anemones and one 1 L water sample were taken from each tank.

1 Dungan AM, Hartman LM, Tortorelli G, Belderock R, Lamb AM, Pisan L et al. *Exaiptasia diaphana* from the Great Barrier Reef: a valuable resource for coral symbiosis research. Symbiosis. 2020; doi:10.1007/s13199-020-00665-0
