# Supplementary material for: Microbiota characterization of Exaiptasia diaphana from the Great Barrier Reef
Source: Anim Microbiome. 2020 Apr 5;2:10. doi: 10.1186/s42523-020-00029-5 (PMC7807684; doi:10.1186/s42523-020-00029-5)
Supplement: Supplementary file 2 — Additional file 2: Figure S4. Holding tank at AIMS. Figure S5. Holding tank outflow with anemones. [file 42523_2020_29_MOESM2_ESM.docx]

**Additional File 2**

Wild proxy anemone acquisition

In October 2016, five anemone polyps were collected as wild proxies of *Exaiptasia diaphana* from the outflow of a 4000 L outdoor holding tank containing live corals, snails, sea cucumbers and fish at the AIMS SeaSim, Townsville, Australia (Figures S4, S5). The tank received seawater from the SeaSim recirculation system with inline 0.45 µm filtration and fractionation to remove particulates and protein respectively. The water temperature was stable at 27 °C, and 120 mL of *Artemia* nauplii feedstock was added daily.


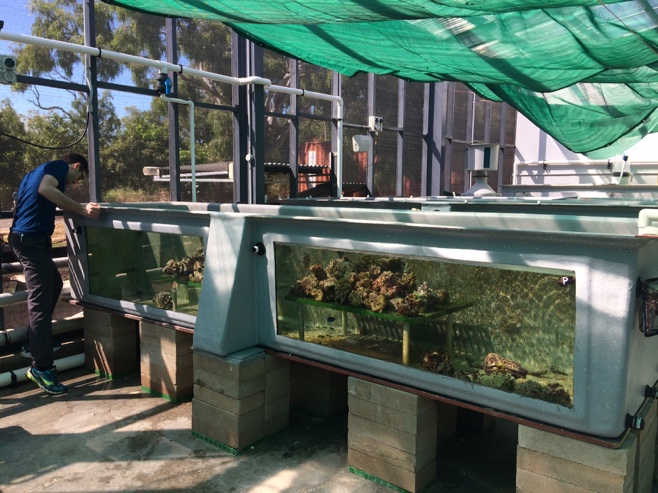

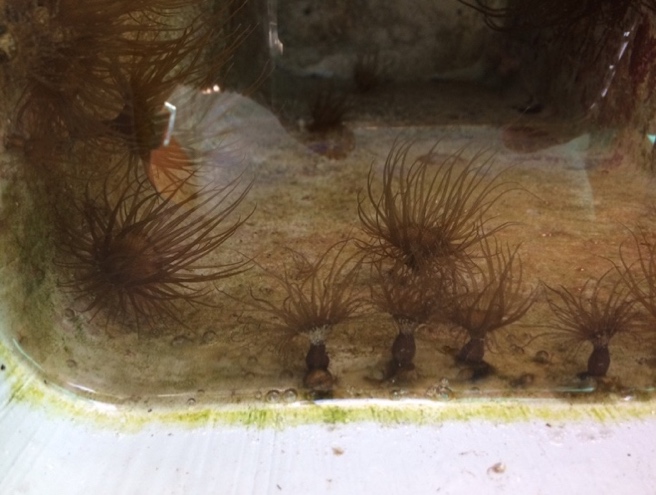


5 cm

Figure S4: Holding tank at AIMS. Figure S5: Holding tank outflow with
 anemones.

For anemone species verification, *E. diaphana*-specific 18S rRNA gene primers (18S_NA [5’ TAAGCACTTGT CTGTGAAACTGCGA 3’]; 18S_NB [5’ AGGAGTCCTCACTAAACCAT 3’] [1]) were used to generate amplicons by single PCRs on DNA extracts (See main text, Methods). The PCR product was checked by 1% agar gel electrophoresis, purified (Bioline II PCR and Gel Kit BIO-52059), diluted (5 ng/µL), and sent to the Australian Genome Research Facility, Melbourne, for Sanger sequencing using the external 18S PCR primers described above, and four internal 18S rRNA gene primers: 18S_NL [5’ AACAGCCCGGTCAGTAACACG 3’]; 18S_NC [5’ AATAACAATACAGGGCTTTTCTAAGTC 3’]; 18S_NY [5’ GCCTTCCTGACTTTGGTTGAA 3’]; 18S_NO [5’ AGTGTTATTGGATGACCTCTTTGGC 3’] [1]. The sequence data was imported into Geneious (v 10.0.4) [2]. The chromatograms were visually inspected, and the sequences manually trimmed then do novo assembled. The top BLAST [3] hits for the consensus sequences against the NCBI database [4] were for *Aiptasia pulchella* or *E. pallida*. However, as *A. pulchella* is synonymous with *E. pallida* [5], and *Exaiptasia diaphana* is the currently approved nomenclature for these anemones [6], all samples were designated *E. diaphana*. The assembled 18S data are available under NCBI BioProject PRJNA575811.

References

1 Grajales A, Rodríguez E. Elucidating the evolutionary relationships of the Aiptasiidae, a widespread cnidarian–dinoflagellate model system (Cnidaria: Anthozoa: Actiniaria: Metridioidea). Mol Phylogen Evol. 2015;94:252-63.

2 Kearse M, Moir R, Wilson A, Stones-Havas S, Cheung M, Sturrock S et al. Geneious Basic: an integrated and extendable desktop software platform for the organization and analysis of sequence data. Bioinformatics. 2012;28:1647-49.

3 Altschul SF, Gish W, Miller W, Myers EW, Lipman DJ. Basic local alignment search tool. J Mol Biol. 1990;215:403-10.

4 Federhen S. The NCBI taxonomy database. Nucleic Acids Res. 2012;40:D136-D43.

5 Grajales A, Rodríguez E. Morphological revision of the genus *Aiptasia* and the family Aiptasiidae (Cnidaria, Actinaria, Metridioidea). Zootaxa. 2014;3826:55-100.

6 Grajales A, Rodríguez E. Case 3790 – Proposed review of Opinion 2404 and reconsideration of Case 3633: *Dysactis pallida* Agassiz in Verrill, 1864 (currently *Exaiptasia pallida*; Cnidaria, Anthozoa, Hexacorallia, Actiniaria): proposed precedence over *Exaiptasia diaphana* (Rapp, 1829). Bull Zool Nomencl. 2019;76:127-31.
