# Supplementary material for: Microbiota characterization of Exaiptasia diaphana from the Great Barrier Reef
Source: Anim Microbiome. 2020 Apr 5;2:10. doi: 10.1186/s42523-020-00029-5 (PMC7807684; doi:10.1186/s42523-020-00029-5)
Supplement: Supplementary file 3 — Additional file 3: Figure S6. Rarefaction curves for all samples. Figure S7. Relative abundance of family level taxa. Figure S8. Legend of family-level taxa. [file 42523_2020_29_MOESM3_ESM.docx]

**Additional file 3**

Figure S6: Rarefaction curves for all samples. Curves for bacterial sequences from all anemone and water samples plateaued, indicating that sequencing depth was sufficient to capture bacterial species diversity (sub-sampling level: 12 000; step: 600).

Table S1: Contaminant ASVs removed from the dataset. Seven ASVs potentially introduced during sample processing were identified with decontam [1] using data from the negative control samples. All were removed from the dataset.

|  | Phylum | Class | Order | Family | Genus | AIMS1-4 anemones  Rel. abundance (%) | AIMS1-4 water  Rel. abundance (%) | Wild proxy anemones  Rel. abundance (%) |
| --- | --- | --- | --- | --- | --- | --- | --- | --- |
| 1 | Proteobacteria | Alphaproteobacteria | Caulobacterales | Caulobacteraceae | *Brevundimonas* | 0.0033 | 0.0000 | 0.0478 |
| 2 | Proteobacteria | Alphaproteobacteria | Sphingomonadales | Sphingomonadaceae | *Sphingomonas* | 0.0063 | 0.0000 | 0.0000 |
| 3 | Proteobacteria | Alphaproteobacteria | Sphingomonadales | Sphingomonadaceae | *Sphingomonas* | 0.0041 | 0.0000 | 0.0000 |
| 4 | Planctomycetes | Planctomycetacia | Pirellulales | Pirellulaceae | *Rhodopirellula* | 0.0001 | 0.0000 | 0.0408 |
| 5 | Actinobacteria | Actinobacteria | Micrococcales | Micrococcaceae | Unclassified | 0.0058 | 0.0000 | 0.0000 |
| 6 | Proteobacteria | Gammaproteobacteria | Pseudomonadales | Moraxellaceae | *Enhydrobacter* | 0.0051 | 0.0000 | 0.0000 |
| 7 | Proteobacteria | Gammaproteobacteria | Betaproteobacteriales | Burkholderiaceae | *Ralstonia* | 0.0350 | 0.0699 | 1.9880 |
|  |  |  |  |  |  | Total (%):  0.0597 | Total (%)  0.0699 | Total (%):  2.0766 |

Family-level bar-charts revealed three samples with overrepresented ASVs (Figure S7). The wild proxy “prox3” and AIMS3 “e33a” samples contained ~25% and ~57% *Enterobacteriaceae* respectively, compared to 0.5%, on average, for the other anemone samples. *Enterobacteriaceae* was absent from the *Artemia* feedstock, therefore it was assumed to be a contaminant. The AIMS4 sample “e43f” contained a high proportion of *Vibrionaceae* (~39%), which were further identified as *Vibrio* sp.. Although *Vibrio* sp. are known coral associates, all other anemone samples contained <4% *Vibrionaceae*, and most <1%. Consequently, prox3, e33a and e43f were removed from the analysis.

Figure S7: Relative proportions of family level taxa. ▲ Red triangles indicate samples deemed contaminated. Control samples were omitted. Bars do not reach 100% because some ASVs could not be classified to family level.

Figure S8: Legend of family-level taxa.

References

1 Davis NM, Proctor DM, Holmes SP, Relman DA, Callahan BJ. Simple statistical identification and removal of contaminant sequences in marker-gene and metagenomics data. Microbiome. 2018; doi:10.1186/s40168-018-0605-2
