# Supplementary material for: Microbiota characterization of Exaiptasia diaphana from the Great Barrier Reef
Source: Anim Microbiome. 2020 Apr 5;2:10. doi: 10.1186/s42523-020-00029-5 (PMC7807684; doi:10.1186/s42523-020-00029-5)
Supplement: Supplementary file 4 — Additional file 4: Table S2. Tukey’s HSD p-values from pair-wise Shannon value comparison. [file 42523_2020_29_MOESM4_ESM.docx]

**Additional file 4**

According to Levene’s test, the variance of the Shannon data for the sample types did not differ significantly from homogeneity: (F_6, 77_ = 0.93, p = 0.48). However, tests for normality by Shapiro-Wilk suggested the data for three sample types, AIMS1 (w = 0.87, p = 0.02), AIMS4 (w = 0.89, p = 0.04) and the wild proxies (w = 0.70, p = 0.01), were not normally distributed. Inspection of the plotted residuals showed that AIMS1 and AIMS4 did not deviate substantially from normality but contained one and two outliers, respectively. The wild proxy result was likely exacerbated by the low number of samples (four). As ANOVA is robust to non-normality [1], the data was analysed by ANOVA and Tukey’s HSD.

Table S2: Tukey’s HSD p-values from pair-wise Shannon value comparison. Values indicating significantly different pairs (𝛼 = 0.05) are highlighted.

|  | AIMS1 | AIMS2 | AIMS3 | AIMS4 | Wild proxies | CC7 |
| --- | --- | --- | --- | --- | --- | --- |
| AIMS2 | 0.116 |  |  |  |  |  |
| AIMS3 | 0.642 | 0.956 |  |  |  |  |
| AIMS4 | 0.999 | 0.077 | 0.521 |  |  |  |
| Wild proxies | 0.000 | 0.000 | 0.000 | 0.000 |  |  |
| CC7 | 0.000 | 0.060 | 0.010 | 0.000 | 0.000 |  |
| H2 | 0.000 | 0.000 | 0.000 | 0.000 | 0.000 | 0.000 |

References

1 Blanca MJ, Alarcon R, Arnau J, Bono R, Bendayan R. Non-normal data: is ANOVA still a valid option? Psicothema. 2017;29:552-57.
