# Supplementary material for: Microbiota characterization of Exaiptasia diaphana from the Great Barrier Reef
Source: Anim Microbiome. 2020 Apr 5;2:10. doi: 10.1186/s42523-020-00029-5 (PMC7807684; doi:10.1186/s42523-020-00029-5)
Supplement: Supplementary file 5 — Additional file 5: Table S3. Relative abundance of phyla in each AIMS1–4 genotype. [file 42523_2020_29_MOESM5_ESM.docx]

**Additional file 5**

Table S3: Relative abundance of phyla in each AIMS1-4 genotype. Empty cell = not detected.

| Phylum | AIMS1  (%) | AIMS2  (%) | AIMS3  (%) | AIMS4  (%) |
| --- | --- | --- | --- | --- |
| *Proteobacteria* | 75.89 | 75.08 | 77.19 | 76.80 |
| *Bacteroidetes* | 17.05 | 13.78 | 18.47 | 13.35 |
| *Spirochaetes* | 0.04 | 5.73 | 0.02 | 4.13 |
| *Planctomycetes* | 1.65 | 2.43 | 1.50 | 1.93 |
| *Acidobacteria* | 1.88 | 1.53 | 1.36 | 2.50 |
| *Chlamydiae* | 2.63 | 0.60 | 0.81 | 0.60 |
| *Actinobacteria* | 0.69 | 0.58 | 0.42 | 0.60 |
| *Firmicutes* | 0.15 | 0.15 | 0.20 | 0.06 |
| *Calditrichaeota* | <0.01 | 0.07 | <0.01 | 0.01 |
| *Verrucomicrobia* | <0.01 | 0.02 | 0.01 |  |
| *Gemmatimonadetes* | <0.01 | <0.01 | 0.02 |  |
| *Cyanobacteria* | 0.01 | 0.02 |  | <0.01 |
| *Dependentiae* |  | 0.01 | 0.01 | <0.01 |
| *Patescibacteria* | <0.01 |  | <0.01 | 0.00 |
| *WPS-2* | <0.01 | <0.01 |  |  |
| *Elusimicrobia* |  | <0.01 |  | <0.01 |
| *Lentisphaerae* |  |  | <0.01 | <0.01 |
| *Fusobacteria* | <0.01 |  |  |  |
