# Supplementary material for: Microbiota characterization of Exaiptasia diaphana from the Great Barrier Reef
Source: Anim Microbiome. 2020 Apr 5;2:10. doi: 10.1186/s42523-020-00029-5 (PMC7807684; doi:10.1186/s42523-020-00029-5)
Supplement: Supplementary file 6 — Additional file 6: Table S4. Pairwise log2 fold change for 20 most abundant phylum-level taxa. Table S5. Pairwise log2 fold change for 20 most abundant genus-level taxa. Figure S9. Heatmaps of the top 20 taxa by relative abundance at order (a) and family (b) levels. Table S6. Pairwise log2 fold change for 20 most abundant order-level taxa. Table S7. Pairwise log2 fold change for 20 most abundant family-level taxa. [file 42523_2020_29_MOESM6_ESM.docx]

**Additional file 6**

Table S4: Pairwise log_2_ fold change (L_2_FC) Table S5: Pairwise log_2_ fold change (L_2_FC)
for 20 most abundant phylum-level taxa for 20 most abundant genus-level taxa
(𝛼 = 0.05). Only results with L_2_FC > 2.00 (𝛼 = 0.05). Only results with L_2_FC > 2.00
are shown. are shown.

| Class | pair | log_2_FoldChange | adjusted p-value |
| --- | --- | --- | --- |
| *Acidimicrobiia* | AIMS1 v WP | -5.25 | 1.39E-05 |
|  | AIMS2 v WP | -4.84 | 3.27E-05 |
|  | AIMS3 v WP | -5.76 | 6.12E-07 |
|  | AIMS4 v WP | -5.26 | 5.16E-06 |
| *Acidobacteriia* | AIMS2 v AIMS4 | 6.91 | 1.35E-02 |
|  | AIMS1 v WP | 2.24 | 1.85E-02 |
|  | AIMS2 v WP | 2.34 | 1.08E-02 |
|  | AIMS4 v WP | 2.39 | 4.34E-03 |
| *BD2-11 terrestrial group* | AIMS1 v WP | -8.01 | 2.88E-02 |
|  | AIMS4 v WP | -9.39 | 4.34E-03 |
| *Holophagae* | AIMS1 v AIMS4 | -3.24 | 3.34E-04 |
|  | AIMS1 v WP | 3.82 | 1.99E-02 |
|  | AIMS2 v WP | 5.85 | 8.20E-05 |
|  | AIMS3 v WP | 4.98 | 1.73E-03 |
|  | AIMS4 v WP | 7.06 | 2.71E-06 |
| *Ignavibacteria* | AIMS1 v WP | -9.27 | 4.57E-02 |
| *Pla3 lineage* | AIMS1 v AIMS2 | -10.39 | 3.21E-31 |
|  | AIMS1 v AIMS3 | -8.89 | 3.86E-22 |
|  | AIMS1 v AIMS4 | -7.98 | 4.63E-18 |
|  | AIMS2 v AIMS4 | 2.41 | 1.98E-02 |
|  | AIMS2 v WP | 9.48 | 6.62E-08 |
|  | AIMS3 v WP | 7.98 | 6.10E-06 |
|  | AIMS4 v WP | 7.06 | 4.94E-05 |
| *Spirochaetia* | AIMS1 v AIMS2 | -10.05 | 7.42E-44 |
|  | AIMS1 v AIMS3 | 3.05 | 8.20E-03 |
|  | AIMS1 v AIMS4 | -9.41 | 1.83E-37 |
|  | AIMS1 v WP | -4.19 | 4.28E-03 |
|  | AIMS2 v AIMS3 | 13.10 | 1.78E-53 |
|  | AIMS2 v WP | 5.86 | 2.44E-06 |
|  | AIMS3 v AIMS4 | -12.46 | 1.74E-47 |
|  | AIMS3 v WP | -7.24 | 1.30E-07 |
|  | AIMS4 v WP | 5.22 | 2.54E-05 |
| *Subgroup 22* | AIMS1 v AIMS2 | 7.26 | 9.39E-18 |
|  | AIMS1 v AIMS4 | 7.11 | 7.85E-17 |
|  | AIMS1 v WP | 4.45 | 5.48E-03 |
|  | AIMS2 v AIMS3 | -6.75 | 8.40E-15 |
|  | AIMS3 v AIMS4 | 6.60 | 5.52E-14 |
|  | AIMS3 v WP | 3.93 | 1.62E-02 |
| *Thermoanaerobaculia* | AIMS2 v WP | -3.86 | 1.32E-02 |
|  | AIMS3 v WP | -4.30 | 7.22E-03 |
|  | AIMS4 v WP | -4.14 | 4.34E-03 |

| Genus | pair | log_2_FoldChange | adjusted p-value |
| --- | --- | --- | --- |
| *Aestuariibacter* | AIMS2 v WP | 4.32 | 1.26E-03 |
|  | AIMS4 v WP | 2.67 | 4.62E-02 |
| *Alteromonas* | AIMS1 v WP | 10.66 | 1.56E-16 |
|  | AIMS2 v WP | 10.90 | 2.48E-17 |
|  | AIMS3 v WP | 10.73 | 7.18E-17 |
|  | AIMS4 v WP | 11.31 | 7.98E-19 |
| *Chitinophagales (uncultured)* | AIMS3 v WP | 2.64 | 1.09E-02 |
| *Cyclobacteriaceae (uncultured)* | AIMS1 v WP | -8.15 | 4.20E-03 |
|  | AIMS2 v WP | -9.52 | 6.38E-04 |
|  | AIMS3 v WP | -11.54 | 1.63E-05 |
|  | AIMS4 v WP | -11.13 | 2.37E-05 |
| *Labrenzia* | AIMS3 v WP | 2.01 | 1.88E-02 |
| *Leisingera* | AIMS1 v WP | 9.18 | 3.98E-13 |
|  | AIMS2 v WP | 9.62 | 1.62E-14 |
|  | AIMS3 v WP | 9.94 | 1.34E-15 |
|  | AIMS4 v WP | 10.54 | 1.28E-17 |
| *Marinobacter* | AIMS1 v WP | 6.21 | 7.37E-21 |
|  | AIMS2 v WP | 6.68 | 2.47E-24 |
|  | AIMS3 v WP | 6.93 | 3.31E-26 |
|  | AIMS4 v WP | 6.42 | 9.72E-23 |
| *Methylotenera* | AIMS1 v AIMS3 | 2.35 | 1.07E-03 |
|  | AIMS1 v WP | 9.42 | 1.04E-09 |
|  | AIMS2 v AIMS4 | -2.58 | 1.94E-04 |
|  | AIMS2 v WP | 7.99 | 3.53E-07 |
|  | AIMS3 v AIMS4 | -3.51 | 1.50E-07 |
|  | AIMS3 v WP | 7.06 | 7.51E-06 |
|  | AIMS4 v WP | 10.57 | 2.18E-12 |
| *Nonlabens* | AIMS1 v WP | 4.53 | 3.10E-03 |
|  | AIMS2 v WP | 4.62 | 2.24E-03 |
|  | AIMS3 v WP | 4.59 | 1.94E-03 |
|  | AIMS4 v WP | 5.39 | 1.50E-04 |
| *Peredibacter* | AIMS4 v WP | 2.23 | 3.54E-03 |
| *Rhodobacteraceae (uncultured)* | AIMS1 v WP | -3.92 | 8.54E-08 |
|  | AIMS2 v WP | -2.61 | 8.11E-04 |
|  | AIMS3 v WP | -4.40 | 1.01E-09 |
|  | AIMS4 v WP | -3.92 | 5.12E-08 |
| *Ruegeria* | AIMS1 v AIMS2 | 5.44 | 6.30E-04 |
|  | AIMS1 v WP | -6.37 | 1.87E-02 |
|  | AIMS2 v WP | -11.81 | 1.09E-06 |
|  | AIMS3 v WP | -9.04 | 2.83E-04 |
|  | AIMS4 v WP | -9.06 | 1.63E-04 |
| *Sedimentitalea* | AIMS1 v WP | 4.43 | 2.29E-08 |
|  | AIMS2 v AIMS4 | 2.12 | 6.94E-06 |
|  | AIMS2 v WP | 5.69 | 1.19E-13 |
|  | AIMS3 v WP | 4.28 | 4.99E-08 |
|  | AIMS4 v WP | 3.56 | 7.73E-06 |
| *Spirochaeta 2* | AIMS1 v AIMS2 | -10.28 | 3.55E-38 |
|  | AIMS1 v AIMS3 | 2.75 | 2.06E-02 |
|  | AIMS1 v AIMS4 | -9.80 | 3.67E-34 |
|  | AIMS1 v WP | -5.73 | 4.83E-05 |
|  | AIMS2 v AIMS3 | 13.03 | 1.13E-46 |
|  | AIMS2 v WP | 4.55 | 1.36E-03 |
|  | AIMS3 v AIMS4 | -12.55 | 5.32E-43 |
|  | AIMS3 v WP | -8.48 | 2.44E-09 |
|  | AIMS4 v WP | 4.07 | 3.13E-03 |
| *Thalassobius* | AIMS1 v WP | 2.37 | 8.67E-11 |
|  | AIMS2 v WP | 2.35 | 7.39E-11 |
|  | AIMS3 v WP | 2.76 | 9.14E-15 |
|  | AIMS4 v WP | 2.21 | 9.57E-10 |

Figure S9: Heatmaps of the top 20 AIMS1-4 taxa by relative abundance at order (a) and family (b) levels. WP = Wild Proxies.

The relative abundance of the 20 most abundant order and family-level taxa was generally consistent between the AIMS1-4 genotypes with the exception of Spirochaetales-Spirochaetaceae, which featured strongly in AIMS2 and AIMS4 only. As described in Additional file 1, this difference may be related to the different culture histories of the AIMS1 and AIMS3 versus AISM2 and AIMS4.

Table S6: Pairwise log_2_ fold change (L_2_FC) Table S7: Pairwise log_2_ fold change (L_2_FC)
for 20 most abundant order-level taxa for 20 most abundant family-level taxa
(𝛼 = 0.05). Only results with L_2_FC > 2.00 (𝛼 = 0.05). Only results with L_2_FC > 2.00
are shown. are shown.

| Order | pair | log2FoldChange | adjusted p-value |
| --- | --- | --- | --- |
| *Acanthopleuribacterales* | AIMS1 v AIMS4 | -2.69 | 7.40E-03 |
|  | AIMS1 v WP | 3.99 | 1.60E-02 |
|  | AIMS2 v WP | 5.52 | 4.73E-04 |
|  | AIMS3 v WP | 4.84 | 2.83E-03 |
|  | AIMS4 v WP | 6.68 | 1.62E-05 |
| *Alteromonadales* | AIMS1 v WP | 3.26 | 1.02E-17 |
|  | AIMS2 v WP | 3.75 | 1.35E-23 |
|  | AIMS3 v WP | 3.51 | 2.44E-20 |
|  | AIMS4 v WP | 3.36 | 5.78E-19 |
| *Betaproteobacteriales* | AIMS1 v WP | 3.17 | 1.51E-03 |
|  | AIMS2 v WP | 2.97 | 1.98E-03 |
|  | AIMS3 v AIMS4 | -2.26 | 8.38E-05 |
|  | AIMS4 v WP | 3.93 | 2.58E-05 |
| *Chlamydiales* | AIMS1 v AIMS2 | 2.03 | 8.10E-10 |
| *Cytophagales* | AIMS1 v AIMS2 | 2.06 | 3.27E-04 |
|  | AIMS1 v WP | -2.12 | 3.84E-02 |
|  | AIMS2 v WP | -4.18 | 8.27E-06 |
|  | AIMS3 v WP | -3.13 | 1.23E-03 |
|  | AIMS4 v WP | -3.17 | 7.50E-04 |
| *Ectothiorhodospirales* | AIMS2 v AIMS4 | 3.86 | 2.65E-03 |
|  | AIMS3 v AIMS4 | 3.26 | 1.43E-02 |
| *Oceanospirillales* | AIMS1 v WP | 3.60 | 5.94E-08 |
|  | AIMS2 v WP | 3.10 | 4.94E-06 |
|  | AIMS3 v WP | 3.21 | 2.05E-06 |
|  | AIMS4 v WP | 3.41 | 3.94E-07 |
| *Oligoflexales* | AIMS1 v WP | 2.22 | 7.12E-03 |
| *Rhodospirillales* | AIMS1 v WP | 2.62 | 1.19E-03 |
|  | AIMS2 v WP | 2.81 | 2.61E-04 |
|  | AIMS3 v WP | 2.74 | 4.15E-04 |
|  | AIMS4 v WP | 3.03 | 5.75E-05 |
| *Spirochaetales* | AIMS1 v AIMS2 | -9.74 | 1.39E-41 |
|  | AIMS1 v AIMS3 | 3.14 | 8.90E-03 |
|  | AIMS1 v AIMS4 | -9.09 | 1.25E-35 |
|  | AIMS1 v WP | -4.59 | 8.15E-04 |
|  | AIMS2 v AIMS3 | 12.88 | 1.87E-52 |
|  | AIMS2 v WP | 5.15 | 4.08E-05 |
|  | AIMS3 v AIMS4 | -12.23 | 7.08E-47 |
|  | AIMS3 v WP | -7.73 | 9.44E-09 |
|  | AIMS4 v WP | 4.50 | 3.49E-04 |

| Family | pair | log2FoldChange | adjusted p-value |
| --- | --- | --- | --- |
| *Alteromonadaceae* | AIMS2 v WP | 2.77 | 1.09E-07 |
|  | AIMS4 v WP | 2.44 | 2.20E-06 |
| *Cryomorphaceae* | AIMS1 v WP | -3.05 | 9.19E-05 |
|  | AIMS2 v WP | -2.04 | 2.16E-02 |
|  | AIMS3 v WP | -2.44 | 2.04E-03 |
|  | AIMS4 v WP | -2.78 | 3.17E-04 |
| *Cyclobacteriaceae* | AIMS1 v WP | -2.85 | 6.18E-03 |
|  | AIMS2 v WP | -4.76 | 1.19E-06 |
|  | AIMS3 v WP | -3.63 | 3.83E-04 |
|  | AIMS4 v WP | -3.47 | 4.44E-04 |
| *Flavobacteriaceae* | AIMS1 v WP | -2.09 | 2.60E-02 |
|  | AIMS2 v WP | -2.11 | 3.35E-02 |
| *Halieaceae* | AIMS1 v WP | -2.49 | 1.49E-03 |
|  | AIMS3 v WP | -2.51 | 1.16E-03 |
| *Marinobacteraceae* | AIMS1 v WP | 6.48 | 1.29E-23 |
|  | AIMS2 v WP | 6.76 | 1.37E-25 |
|  | AIMS3 v WP | 7.16 | 7.39E-29 |
|  | AIMS4 v WP | 6.46 | 1.07E-23 |
| *Methylophilaceae* | AIMS1 v WP | 9.97 | 1.68E-13 |
|  | AIMS2 v AIMS4 | -2.57 | 4.04E-07 |
|  | AIMS2 v WP | 8.40 | 1.97E-09 |
|  | AIMS3 v AIMS4 | -2.73 | 7.00E-08 |
|  | AIMS3 v WP | 8.24 | 2.19E-09 |
|  | AIMS4 v WP | 10.97 | 1.67E-16 |
| *Nannocystaceae* | AIMS1 v WP | 2.12 | 4.50E-03 |
|  | AIMS3 v WP | 2.33 | 1.34E-03 |
| *Rhizobiaceae* | AIMS1 v WP | -2.50 | 2.60E-02 |
| *Spirochaetaceae* | AIMS1 v AIMS2 | -9.96 | 7.42E-40 |
|  | AIMS1 v AIMS3 | 3.10 | 6.44E-03 |
|  | AIMS1 v AIMS4 | -9.22 | 1.04E-33 |
|  | AIMS1 v WP | -5.24 | 1.15E-04 |
|  | AIMS2 v AIMS3 | 13.06 | 1.47E-50 |
|  | AIMS2 v WP | 4.72 | 6.49E-04 |
|  | AIMS3 v AIMS4 | -12.32 | 1.51E-44 |
|  | AIMS3 v WP | -8.34 | 1.65E-09 |
|  | AIMS4 v WP | 3.98 | 2.77E-03 |
| *Stappiaceae* | AIMS1 v WP | 2.23 | 6.18E-03 |
|  | AIMS3 v WP | 2.19 | 6.54E-03 |
