# Supplementary material for: Microbiota characterization of Exaiptasia diaphana from the Great Barrier Reef
Source: Anim Microbiome. 2020 Apr 5;2:10. doi: 10.1186/s42523-020-00029-5 (PMC7807684; doi:10.1186/s42523-020-00029-5)
Supplement: Supplementary file 7 — Additional file 7: Table S8. Output from a GLM-based analysis comparing the AIMS1–4 bacterial community compositions (‘tank’ nested within ‘genotype’). Table S9. Output from a GLM-based analysis comparing the AIMS1–4 bacterial community compositions (‘tank’ and ‘genotype’ as separate main effects). [file 42523_2020_29_MOESM7_ESM.docx]

**Additional File 7**

Table S8: Output from a GLM comparing the AIMS1-4 bacterial community compositions (‘tank’ nested within ‘genotype’).

Analysis of Deviance Table

Model: manyglm(formula = data ~ data$genotype/data$tank, family = "negative_binomial")

Multivariate test:

Res.Df Df.diff Dev Pr(>Dev)

(Intercept) 69

combo$ genotype 66 3 2434 0.001 ***

combo$genotype:combo$tank 58 12 3506 0.001 ***

Signif. codes: 0 ‘***’ 0.001 ‘**’ 0.01 ‘*’ 0.05 ‘. 0.1 ‘ ’ 1

Table S9: Output from a GLM comparing the AIMS1-4 bacterial community compositions (‘tank’ and ‘genotype’ as separate main effects).

Analysis of Deviance Table

Model: manyglm(formula = data ~ data$tank + data$genotype, family = "negative_binomial")

Multivariate test:

Res.Df Df.diff Dev Pr(>Dev)

(Intercept) 69

combo$tank 58 11 5941 0.001 ***

combo$genotype 58 3 0 0.997

Signif. codes: 0 ‘***’ 0.001 ‘**’ 0.01 ‘*’ 0.05 ‘. 0.1 ‘ ’ 1
